# Protocol of an Exploratory Single-Arm Study to Evaluate the Safety and Immunogenicity of KD-414 as a Booster Vaccine for SARS-CoV-2 in Healthy Adults (KAPIVARA)

**DOI:** 10.3390/life12070966

**Published:** 2022-06-27

**Authors:** Yuriko Terayama, Noriko Tomita, Junko Terada-Hirashima, Yukari Uemura, Yosuke Shimizu, Junko S. Takeuchi, Yuki Takamatsu, Kenji Maeda, Ayako Mikami, Mugen Ujiie, Wataru Sugiura

**Affiliations:** 1 Respiratory Medicine, National Center for Global Health and Medicine, Tokyo 162-8655, Japan; yhorikawa@hosp.ncgm.go.jp; 2 Center for Clinical Sciences, National Center for Global Health and Medicine, 1-21-1 Toyama, Shinjuku-ku, Tokyo 162-8655, Japan; ntomita@hosp.ncgm.go.jp (N.T.); yuemura@hosp.ncgm.go.jp (Y.U.); yshimizu@hosp.ncgm.go.jp (Y.S.); jtakeuchi@hosp.ncgm.go.jp (J.S.T.); amikami@hosp.ncgm.go.jp (A.M.); wsugiura@hosp.ncgm.go.jp (W.S.); 3 Department of Refractory Viral Infections, National Center for Global Health and Medicine Research Institute, Tokyo 162-8655, Japan; ytakamatsu@ri.ncgm.go.jp (Y.T.); kmaeda@ri.ncgm.go.jp (K.M.); 4 Disease Control and Prevention Center, National Center for Global Health and Medicine, Tokyo 162-8655, Japan; mgujiie@hosp.ncgm.go.jp

**Keywords:** COVID-19, KD-414, inactivated vaccine, booster

## Abstract

Background: The coronavirus disease 2019 (COVID-19) pandemic is currently ongoing, and there have been significant efforts in the development of COVID-19 vaccines. However, the neutralizing antibody titers in vaccinated individuals are reported to progressively decrease over time. Japanese pharmaceutical companies have published the results of Phase I and II studies on the safety and efficacy of different vaccines. Final clinical trials will be conducted with the aim of practical application by March 2023. To effectively utilize vaccines developed by Japanese companies, the efficacy and safety of a booster dose (i.e., third vaccination) must be evaluated among individuals who have received three doses of different vaccines. Methods: This protocol describes a study that aims to examine the effect of a booster dose of “KD-414”, a novel Japanese inactivated vaccine, on antibody titers among participants involved in a previous study. Volunteers in this protocol will be recruited from participants in the previous study and immunized with KD-414 after obtaining consent. The antibody titers, before and after immunization with KD-414, among participants who previously received two doses of the BNT162b2 mRNA vaccine, will be comparatively analyzed. Discussion: The reactogenicity and immunogenicity of seven different COVID-19 vaccines including an inactivated vaccine as a third dose after two doses of ChAdOx1 nCov-19 or BNT162b2, has been tested previously, and found to be superior to control (quadrivalent meningococcal conjugate vaccine) regardless of which vaccine had been received during the initial course. This suggests that many types of third booster doses are efficacious. It is anticipated that this study will provide evidence of the safety and immunogenicity of KD-414 as a booster vaccine, which will have profound public health implications.

## 1. Background

The coronavirus disease 2019 (COVID-19) vaccine developed by Pfizer-BioNTech, BNT162b2, was approved for emergency use on 14 February 2021 by the Japanese Ministry of Health, Labor and Welfare. Japan’s vaccination program for healthcare workers was started on 17 February 2021. The efficacy of the vaccine is reported to be >90% among individuals who have received two doses of the vaccine [1]. However, the neutralizing antibody titers in vaccinated individuals are reported to progressively decrease over time [2]. The administration of a third booster dose enhanced the neutralizing antibody titers. Based on these findings, the Centers for Disease Control and Prevention (CDC) Advisory Committee of the United States of America recommend a third booster dose for the elderly population (aged ≥ 65 years) and patients with comorbidities who are at high risk of severe COVID-19. The US Food and Drug Administration provided emergency use authorization for booster doses to various individuals, including those involved in high-risk occupations. The Japanese Health, Labor, and Welfare Council also acknowledged the need for booster doses. However, no novel COVID-19 vaccine was approved for booster doses until September 2021 and vaccination eligibility for specific booster doses is to be discussed on an ongoing basis.

Japanese pharmaceutical companies have published the results of Phase I and II studies on the safety and efficacy of different vaccines. Final clinical trials will be conducted with the aim of practical application by March 2023. To effectively utilize vaccines developed by Japanese companies, the efficacy and safety of booster doses must be evaluated among individuals who have received two doses of the BNT162b2 mRNA vaccine. It is important to evaluate the effect of combined vaccination, for which primary immunization was examined in detail, at this time, because the opportunity for evaluation in such a setting will be lost when additional immunization becomes widespread. Hence, it will be important to evaluate the effect of combined vaccination, for which primary immunization was examined in detail, at this time.

In March 2021, at public expense, the National Center for Global Health and Medicine (NCGM) conducted an observational study of 100 healthcare volunteers from NCGM staff vaccinated with the BNT162b2 mRNA vaccine (Pfizer-BioNTech). The antibody titers were monitored for nine months (*A survey of vaccine-induced COVID-19 antibody titer to verify temporal change*; NCGM Ethical Review Board Approval Number: NCGM-A-004175-04). An in-depth evaluation of the primary immunization revealed that the highest antibody titers were observed on day 7 post-second dose (day 28 post-first dose), which gradually declined thereafter [3,4].

A previous study has shown that a third dose with CoronaVac, an inactivated vaccine, given eight months after a second dose with CoronaVac, significantly increased neutralizing antibody concentrations against SARS-CoV-2 in healthy adults aged 18 years and older [5]. Another study investigated the reactogenicity and immunogenicity of seven different COVID-19 vaccines, including inactivated vaccine (VLA2001), as a third dose after two doses of ChAdOx1 nCov-19 or BNT162b2, showing that the immunogenicity of homologous or heterologous third dose booster vaccination with all tested vaccines was superior to control (quadrivalent meningococcal conjugate vaccine) regardless of which vaccine had been received during the initial course [6]. This suggests that many types of third booster dose are efficacious and supports the potential use of a booster dose of “KD-414”, a novel Japanese inactivated vaccine.

KD-414 is an inactivated vaccine against SARS-CoV-2 being developed by KM-Biologics Co., Ltd. (Kumamoto, Japan). It is an inactivated whole-particle vaccine containing aluminum hydroxide added as an immune aid after growth of SARS-CoV-2 on Vero cells from African green monkey kidney and purification and inactivation of the virus. Currently, Phase I/II clinical trials have been completed in Japan and have shown efficacy of KD-414 in adults and the elderly, and that adverse reactions are comparable to general inactivated vaccines with fewer adverse reactions than for mRNA vaccines. It is thought that KD-414 could provide a new option for those who have difficulty with existing vaccinations or who require higher levels of safety.

In a Phase 1/2 study conducted in JAPAN, only one convalescent fever (39 °C or higher) occurred in 29 subjects with severe adverse reaction after administration of KD-414 containing 10 μg of inactivated coronavirus (SARS-CoV-2). The neutralizing antibody GMT at 28 days after the second vaccination was 12.1 (IU equivalent 44.9–63.4 IU/mL) for adults (aged 20–64 years) and 7.3 (IU equivalent: 27.1–38.3 IU/mL) for the elderly (aged 65 years or older). It approximated an estimated neutralizing antibody titer of 54 IU/mL (95% CI 30–96 IU/mL) required to control the onset of disease [7] In addition, the value was higher than the neutralizing antibody titer for which a 70% efficacy rate of other vaccines could be expected [8].

This protocol describes a study that aims to examine the effect of a booster dose with KD-414 on the antibody titers among participants involved in the previous study [1,2]. Consenting volunteers will be immunized with KD-414. The antibody titers, before and after immunization with KD-414, among participants who had previously received two doses of the BNT162b2 mRNA vaccine will be comparatively analyzed. This trial was registered with the Japan Registry of Clinical Trials (Clinical Trial Plan Number: iRCTs031210388; https://jrct.niph.go.jp/latest-detail/jRCTs031210388; first registration date: 22 October 2021).

## 2. Methods

**Information on drugs used in clinical research (test/comparator products)**

**Name of the drug (generic and brand name)**

(1) Test vaccine: KD-414
(2) Dosage form: Intramuscular injection
(3) Properties: Exhibits uniform white turbidity upon shaking
(4) Storage conditions: Maintained at 2–8 °C under light shielding
(5) Composition: Each vial comprises 0.7 mL of KD-414. Each 0.5 mL of KD-414 contains 10 μg of inactivated coronavirus (SARS-CoV-2) as protein content and aluminum hydroxide as adjuvant.

**Dosage administration route and** **volume**

Intramuscular administration with 0.5 mL/inoculation.

**Target population (age group, sex, and disease history)**

Individuals who have already received two doses of the BNT162b2 mRNA vaccine in the previous study (“*Study on the changes in antibody titers by vaccination with a novel coronavirus vaccine*”) will be enrolled in this study.

**Inclusion criteria**

(1) Individuals who have received two doses of BNT162b2 mRNA vaccine and have completed the measurement of antibody titer on Day 29 (Day 7 after the second dose) in the previous study [3,4].
(2) Individuals who provide written informed consent, comply with the instructions during the study period, undergo medical examination specified in the study protocol, and can report symptoms.

**Exclusion criteria**

(1) Individuals who received three doses of the BNT162b2 mRNA vaccine at the time of obtaining informed consent.
(2) Pregnant women, women who are potentially pregnant, women who wish to become pregnant before completing the post-test, and breastfeeding women.
(3) Individuals with fibrodysplasia ossificans progressiva.
(4) Individuals with underlying diseases, such as serious cardiovascular disease, kidney disease, liver disease, blood disease, developmental disorders, respiratory diseases, and diabetes mellitus.
(5) Individuals with convulsion in the past.
(6) Individuals with a previous diagnosis of immunodeficiency and those with relatives with congenital immunodeficiency.
(7) Individuals who may be allergic to the ingredients of KD-414.
(8) Individuals who have participated in another clinical trial within the past four months (120 days) from the date of proposed vaccination with KD-414 and who have received other test substances (excluding placebo), or who are scheduled to participate in another clinical trial during the study period.
(9) Individuals who have received blood transfusions or gamma globulin products within the past three months (90 days) or have received high-dose gamma globulin products (200 or more mg/kg bodyweight) within the past six months (180 days) from the date of proposed vaccination with KD-414.
(10) Patients who have received radiotherapy, immunosuppressive drugs (topical drugs are available), immunosuppressive therapy, antirheumatic drugs, adrenocorticotropic hormones, or corticosteroids that affect immune function within six months (180 days) after the date of vaccination (if the total daily dose of prednisolone was ≥2 mg/kg bodyweight and the treatment duration was ≥14 days, topical drugs may be used).
(11) Individuals immunized with the live vaccine within one month or inactivated vaccine within one week.
(12) Individuals with a history of breakthrough infection who wish to be immunized with KD-414.
(13) Individuals judged by the investigator (or sub-investigator) to be ineligible for the study.

**Individuals requiring caution about vaccination**

Patients who exhibit the pathological conditions listed below will be carefully monitored through medical examination, and their participation in the study will be considered. The patients will be informed about the aims and endpoints of the study, as well as adverse reactions. Informed consent will be obtained considering their health status and constitution before vaccination.

(1) Individuals with underlying diseases, such as cardiovascular disease, kidney disease, liver disease, hematological disease, developmental disorder, respiratory disease, and diabetes mellitus.
(2) Individuals who have experienced fever within two days of vaccination or who have had suspected allergy symptoms, such as a generalized rash (not applicable if it is confirmed that the causative ingredient is not included in KD-414)
(3) Individuals with convulsions in the past.
(4) Individuals who may be allergic to any of the ingredients of KD-414 (thimerosal).

**Criteria for postponement of vaccination and discontinuation of KD-414**

**Criteria for deferring vaccination of test drugs**

Vaccination will be postponed for study subjects who meet any of the following criteria on the scheduled date of vaccination with KD-414. However, if the vaccination deferral criteria are no longer met, vaccination may be performed.

(1) Individuals with fever (above 37.5 °C).
(2) Individuals suffering from serious acute illness.
(3) The investigator or sub-investigator judges the vaccination with KD-414 to be inappropriate.

**Criteria for discontinuation**

The study participation will be discontinued under the following conditions:

(1) Subjects request withdrawal of consent to participate in the study.
(2) An exclusion criterion is not satisfied.
(3) Compliance with the study protocol is not possible.
(4) The study is discontinued.
(5) The investigator or sub-investigator judged the continuation of the study to be difficult.

**Content of clinical research**

Type of clinical trial

Open-label, single-arm study.

**Randomization and blinding**

Not applicable.

**Use of pharmaceuticals and medical devices for study subjects**

**Details of treatment period and observation period (including follow-up)**

The schedule of KD-414 inoculation has been approved by the Clinical Research Review Board. The treatment period is one day, followed by a 40-day observation period.

**Specific methods (including schedules)**

**Figure 1:**
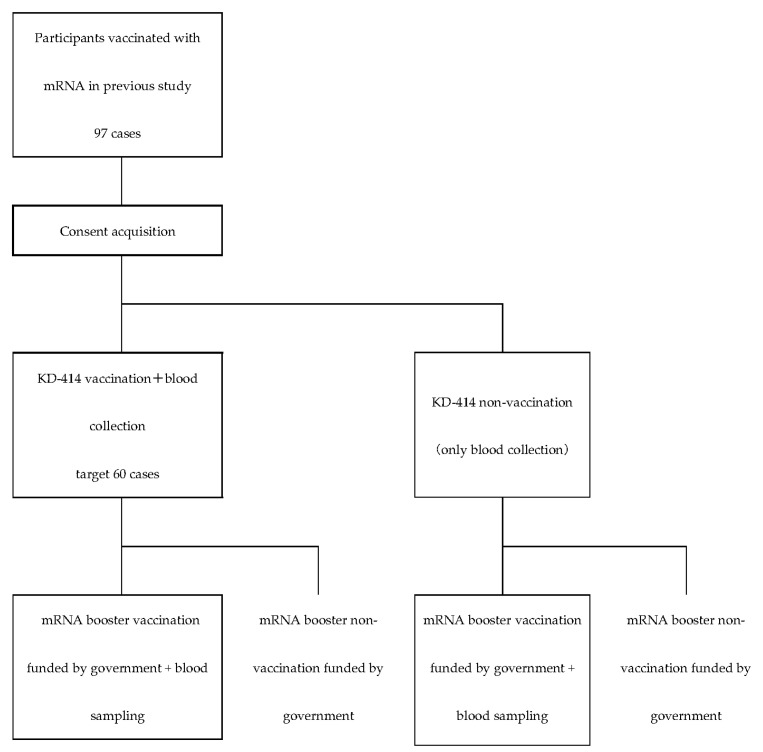
Flowchart of study recruitment process.

The study will be conducted according to the schedules shown in Table 1, Table 2 and Table 3 and the recruitment process is shown in Figure 1.

If a third dose of BNT162b2 mRNA is received in the future at public expense, the immunogenicity-related parameters and adverse events will be recorded on days 0, 7, and 40 post-vaccination.

**Visit 1 (Inclusion of study subjects and KD-414 vaccination/registration)**

(1) The investigator or sub-investigator will explain the test and obtain consent in advance. This is described in further detail in the section titled “Method of obtaining consent for clinical research subjects”.
(2) The investigator or sub-investigator will confirm the background and eligibility of the study subjects and prepare a registration form.

(hereafter, only in the KD-414 group)

(1) The investigator or sub-investigator will collect the blood for immunogenicity evaluation.
(2) The investigator or sub-investigator will conduct physical examination and body temperature measurement for study subjects and inoculate them with KD-414 if the inoculation is judged to be feasible.
(3) Follow-up observation will be performed for approximately 15–30 min after the inoculation of KD-414. In case of an adverse event, the investigator or sub-investigator will take appropriate measures.
(4) The symptoms should alleviate or stabilize if it is judged necessary to continue to confirm the safety due to the occurrence of adverse events.
(5) Study subjects vaccinated with KD-414 will be requested to complete a health diary

**Visits 2 and 3 (post hoc observations)**

(1) The investigator or sub-investigator will collect the blood for immunogenicity evaluation.
(2) The investigator or sub-investigator will examine the study subjects and review the entries of the health diary (Visit 2 only).
(3) In case the adverse event caused by KD-414 has not been resolved, the recovery and stabilization of symptoms must be confirmed (only if adverse events occur/continue).

**Visits 1–3 (public cost booster)**

Investigators will obtain informed consent if booster doses are to be administered to the healthcare workers in the future. Information on vaccinations will be received from the hospital. Personnel who do not receive vaccination in the hospital may be contacted individually.

(1) The investigator or sub-investigator will collect the blood for antibody titration.
(2) Study subjects will receive a publicly funded supplemental coronavirus vaccine during Visit 1.
(3) Study subjects immunized with the vaccine will be requested to complete a health diary.

**Survey items**

**Subject characteristics**

Information from previous study (*A survey of vaccine-induced COVID-19 antibody titer to verify temporal change*; NCGM Ethical Review Board Approval Number: NCGM-A-004175-04) should also be used.

(1) Work career (identification of COVID-19 practice engagement and working contacts with patients with COVID-19)
(2) Attributes of study subjects (age, sex, occupation type, etc.)
(3) Diseases and medications that may affect antibody production or the Th1/Th2 ratio (as appropriate)
(4) Adverse reaction information (fever, malaise, etc.)
(5) Medical examination data (anamnesis, antibody-equivalent to SARS-CoV-2) as appropriate
(6) Content of vaccination questionnaire
(7) History of BNT162b2 mRNA (date of receiving the first and second dose of vaccination)
(8) Data from NCGM health survey after vaccination (conducted as a vaccination project)
(9) Data from NCGM staff antibody surveillance on antibodies against SARS-CoV-2 (this study protocol supplemented with data provision for this study was approved by the Ethics Committee of the Center Hospital of the National Center for Global Health and Medicine, Tokyo, Japan.).

**Investigation items for efficacy endpoints**

(1) COVID-19 disease

**Investigation items for safety endpoints**

(1) Examination and measurement of body temperature
(2) Health diary entry review

The investigator will request the study subjects to record some measurements in a health diary.

① Subaxillary temperature: The investigator measures and records the body temperature daily for seven days after inoculation with KD-414. The maximum body temperature should be recorded if more than one measurement is performed per day.
② Inoculation site reaction: Study subjects must record the presence or absence of an inoculation site reaction daily for seven days after inoculation. In particular, the major axis should be measured and recorded if redness or swelling is observed at the inoculation site.
③ Subjective symptoms/objective findings: The study subjects should record any subjective symptoms or objective findings.

NOTE: If pyrexia is observed seven days after the inoculation, the body temperature measurement should be continued. The date on which the temperature decreases to <37.5 °C should also be recorded.

**Investigation items for immunogenicity endpoints**

(1) Timing of blood sampling: Visits 1 (before vaccine inoculation), 2, and 3.
(2) Measurements:

Determination of neutralizing antibody titers against live SARS-CoV-2 viruses (on days 0, 7, and 40 post-inoculation and in the residual sample collected on days 7 and 40 in the previous studies; conducted at the Department of Refractory Viral Infections, National Center for Global Health and Medicine Research Institute, Tokyo, Japan).

① Determination of neutralizing antibodies (on days 0, 7, and 40 post-inoculation) against live SARS-CoV-2 virus (in residual samples collected on days 7 and 40 in previous studies; conducted by the Department of Refractory Viral Infections, National Center for Global Health and Medicine Research Institute, Tokyo, Japan) is shown in Table 4.

Three assays were performed to detect the IgM and IgG antibodies against SARS-CoV-2 spike protein (IgM-S and IgG-S, respectively) and IgG antibodies against SARS-CoV-2 nucleocapsid protein (IgG-N). The presence of IgG-N antibodies can indicate SARS-CoV-2 infection prior to the study and during follow-up, regardless of the BNT162b2 vaccination status. In contrast, the presence of IgM-S and IgG-S indicates previous infection and/or humoral immunity following BNT162b2 vaccination, because BNT162b2 is constructed to express the full-length spike protein.

② Antibody (IgG) titers against the viral nucleocapsid protein

The Abbott ARCHITECT^®^ SARS-CoV-2 anti-N IgG assay based on semi-quantitative CMIA was performed. Determination by reagent is shown in Table 4 (determination on days 0, 7, and 40 post-inoculation)

③ Antibody (IgM) titers against viral spike proteins

The AdviseDx SARS-CoV-2 IgM assay, based on semi-quantitative CMIA, was performed using the Abbott ARCHITECT^®^ according to the manufacturer’s instructions. Determination by reagent is shown in Table 4.

④ Antibody (IgG) titers against viral spike proteins

The AdviseDx SARS-CoV-2 IgG II assay was performed using the Abbott ARCHITECT^®^ following the manufacturer’s protocol. Determination by reagent is shown in Table 4.

⑤ Flow cytometric analysis of Th1 and Th2 cells (in samples collected on day 7 post-inoculation) (outsourced to SRLs, Tokyo, Japan).

The percentage of Th1 (IFNγ^+^ and IL-4^−^) and Th2 (IFNγ^-^ and IL-4^+^) cells among CD4^+^ lymphocytes will be calculated.

⑥ QuantiFERON SARS-CoV-2 RUO test (QIAGEN, Germany; performed on days 0 and 7 post-inoculation)

SARS-CoV-2 specific CD4^+^ and CD8^+^ T cell responses were investigated. We quantified T cell-produced interferon-gamma (IFN-γ) in response to SARS-CoV-2 spike peptides using QuantiFERON SARS-CoV-2 RUO (QIAGEN, Germany) according to the manufacturer’s instructions.

Volume of blood sampling: 17 mL for Visits 1 and 3 and 22 mL for Visit 2. In total, 56- and 33-mL blood samples were collected from subjects immunized with the study vaccine (KD-414) and unvaccinated subjects, respectively. In total, 56 mL was collected during immunization at public cost.

(1) Treatment method: The blood sample will be allowed to stand at room temperature for 30 min as a guide. After coagulation, the blood samples will be centrifuged at 3000 rpm for 10 min to obtain the serum. The serum will be stored at <−20 °C.
(2) Labeling and transportation methods for storage containers of specimens: Labels will include the name of the study subject, subject identification code, time of blood sampling, and date of blood sampling. The labels will be attached to the sample storage container.

**Evaluation and documentation of adverse events**

(1) The investigator will record the following items on the case report form regarding adverse events in the study subjects from the date of vaccination to Visit 3 (post hoc observations).
(2) Name of the adverse event
(3) Day of adverse event onset
(4) Severity of adverse event (see the section titled “Severity classification of adverse events”).
(5) Seriousness of the adverse event (see the section titled “Reporting of diseases to the certified Clinical Research Review Board (research using unapproved or off-label drugs, etc.);”).
(6) Treatment of the adverse event
(7) Outcome (recovery (including remission), recovery but with sequelae, not recovered, death, unknown), and date of outcome
(8) Causal relationship to the study vaccine (see the section titled “Causal relationship between study drugs and adverse events”).

**Acceptable therapies (including emergency care) before and during the study period**

The concomitant use of drugs required for symptomatic treatment is acceptable in the event of an adverse reaction associated with vaccination.

**Therapies prohibited before and during the** **study period**

(1) Immunization with other COVID 19 vaccines is prohibited for 40 days post-KD-414 vaccination.
(2) Other vaccines are not allowed for 7 days post-KD-414 vaccination.

**Clinical evaluation**

The relevant clinical guidelines from the WHO will be followed in relation to the clinical evaluation of the vaccine [9,10].

**Evaluation items**
**Primary endpoint**

Neutralizing antibody titers against SARS-CoV-2 on day 7 post-immunization

Rationale: Clinical trials evaluating the efficacy of SARS-CoV-2 vaccine candidates in preventing the development of COVID-19 should be conducted. However, a long study period is required to assess the onset of preventive effects, which is not practical as this is an exploratory study. To investigate the need for a large validation study, the immunogenicity-related parameters, including neutralizing antibody titers, will be evaluated as an important endpoint in this study. The primary assessment time point in this study is day 7 post-immunization as the highest titer was observed at this time point in the previous study.

**Secondary endpoint**

<Immunogenicity>

(1) Titers of neutralizing antibodies against SARS-CoV-2 on day 40 post-inoculation
(2) IgG antibodies against SARS-CoV-2 spike protein (IgG-S) on days 7 and 40 post-inoculation
(3) IgG antibodies against SARS-CoV-2 nucleocapsid protein (IgG-N) on days 7 and 40 post-inoculation
(4) IgM antibodies against SARS-CoV-2 spike protein (IgM-S) on days 7 and 40 post-inoculation
(5) Th1 and Th2 responses against SARS-CoV-2 on day 7 post-inoculation
(6) QuantiFERON SARS-CoV-2 RUO test on days 7 and 40 post-inoculation

<Efficacy>

(1) COVID-19 incidence till day 40 post-inoculation

<Safety>

(1) All adverse events occurring between days 0 and 40 post-vaccination, deaths due to adverse events, serious adverse events other than death, significant adverse events, and severe (Grade 3 or higher) adverse events
(2) Physician-reported outcomes
  ① Specific local adverse events *1
  ② Specific systemic adverse events *2
  ③ Unspecified adverse events *3
(3) Maximum body temperature from day 0 to day 6 post-inoculation
(4) Subject-reported outcomes

*1 Specific local adverse events: erythema, swelling, induration, and pain at the injection site between day 0 and day 6 post-inoculation.

*2 Specific systemic adverse events: fever (body temperature of ≥37.5 °C, if present), headache, malaise, nausea, and myalgia between day 0 and day 6 post-inoculation

*3 Unspecified adverse events: Adverse events that do not fall into the category of specified adverse events between day 0 and day 6 post-inoculation are classified as unspecified adverse events. Erythema, swelling, induration, and pain at the injection site, pyrexia, headache, malaise, nausea, and myalgia at day 7 post-inoculation are defined as non-specified adverse events.

**Statistical analysis** **items**
**Analysis sets**
**Study protocol-adapted analysis set (per-protocol set; PPS)**

Among the enrolled participants, subjects associated with major protocol failure and those in whom blood sampling for titration on day 7 post-immunization cannot be performed are excluded.

**Safety set**

All patients who received KD-414 will be included.

**Target sample size and rationale**

Target number of subjects receiving KD-414 to evaluate the main hypothesis: 60 subjects

Target sample size for this study: 97 patients

Rationale: Determining if the antibody titer after the booster dose (KD-414) was lower than that after the second dose of BNT162b2 mRNA. The IgG-S antibody titer after the administration of the second dose of BNT162b2 mRNA vaccine will be investigated because the data for neutralizing antibody titers evaluated using the assay used in this study are not available. The titers of IgG-S and neutralizing antibodies are reported to be highly correlated. We assume that the titer of IgG-S after immunization with KD-414 is similar to that after immunization with the second dose of BNT162b2 mRNA in this study population (4.25 (standard deviation; SD 0.35) on a log_10_ scale). The World Health Organization guidelines on vaccine development set a geometric mean titer (GMT) ratio of 0.67 as the threshold for testing non-inferiority. In the case that the lower limit of the 95% confidence interval for the GMT of IgG-S for the KD-414 to that of Pfizer vaccine is >0.67, the antibody titer after the booster of KD-414 is not inferior to the second dose of BNT162b2 mRNA. Based on these assumptions, the required sample size for 90% power would be 45 for an SD of 0.35, whereas it would be 58 for an SD of 0.4 (Table 5).

The main objective of this study is to obtain consent for immunization with KD-414 from 97 patients with titer test results up to the second dose. Although the rate of informed consent is unknown, the target sample size will be 60 to determine 58 post-vaccination titer values after immunization with KD-414. However, even if the number of subjects enrolled is below 60, a certain power will be assured.

Additionally, patients who do not agree to receive KD-414 (consented to participate in this study) will also be enrolled to compare the antibody titers of non-vaccinated subjects with KD-414 with those of vaccinated subjects. The target sample size for non-vaccinated and vaccinated cases combined will be 97 patients who participated in the previous study.

**Criteria for discontinuation of clinical studies**

(1) Ethical or medical reasons (including those to ensure the safety of study subjects)
(2) Significant or continued non-compliance determined by the investigator or research institution
(3) The Clinical Research Review Board’s decision to terminate or suspend the clinical trial during the continuation review of the ongoing trial
(4) In cases where study subjects are unlikely to satisfy the inclusion criteria
(5) The scientific justification for conducting this study is not satisfactory
(6) The research physician has abandoned the study

**Handling of cases**

The principal investigator and the individual responsible for statistical analysis will decide on the handling of the enrolled cases after discussion. In case of a problem, the investigator and the statistical analysis manager should discuss and decide on the handling of the case. The details of the case-handling decisions will be retained in the record.

**Data handling**

In case of any doubt regarding the handling of data during data collection or analysis, the principal investigator and the statistical analysis manager will decide how to proceed after discussion. No imputation is performed for the missing data. Details, including handling outliers, are specified in the statistical analysis plan.

**Statistical analysis items and analysis plan**

The original version of the analysis plan will be generated prior to data fixation.

**Analysis of study subject background**

Background factors will be tabulated for PPS and KD-414-vaccinated and non-vaccinated cases.

**Primary endpoint analysis**

- Primary analysis

The geometric mean of the neutralizing antibody titers will be calculated for PPS cases on day 7 post-inoculation. Meanwhile, the geometric mean ratio of the neutralizing antibody titer at day 7 post-BNT162b2 mRNA vaccination to the geometric mean value and the 95% confidence interval calculated in the previous study (“*A survey of vaccine-induced COVID-19 antibody titer to verify temporal change*”: NCGM Ethical Review Board Approval Number: NCGM-A-004175-04) will be used.

- Secondary analysis for the primary endpoint
  (1) The geometric mean of the neutralizing antibody titer on day 7 against day 0 will be calculated for PPS cases. Additionally, the geometric mean of neutralizing antibody titers in non-vaccinated subjects on day 40 will be calculated. The geometric mean ratio of neutralizing antibody titers in vaccinated subjects to that in non-vaccinated subjects among PPS cases will also be calculated.
  (2) The percentage of cases in which the geometric mean ratio of neutralizing titers on day 7 to those on day 0 was ≥4 will be calculated.
  (3) Subgroup analyses will be performed on the following items for test drug-vaccinated and non-vaccinated cases among PPS cases:

▪ Work history (presence or absence of work-related contacts)
▪ Sex (male/female)
▪ Age (below or above the median)
▪ Medical history
▪ Interval between the third dose and the second dose of the BNT162b2 mRNA vaccine.
▪ By variant
  (4) Among PPS cases, the geometric mean neutralizing antibody titer on day 7 will be calculated for unvaccinated cases. Additionally, if the non-vaccinated subject received the third dose of other vaccines, the geometric mean of neutralizing antibodies on day 7 after the third dose of other vaccines will be calculated.
  (5) Among PPS cases, the geometric mean neutralizing antibody titers on day 7 will be calculated for unvaccinated and vaccinated cases and compared using the Wilcoxon rank-sum test. Additionally, the geometric mean ratio of neutralizing antibodies on day 7 post-third dose of other vaccines against the vaccinated subject and the 95% confidence interval will be calculated for non-vaccinated subjects.

**Analysis of the secondary variables**

(1) Neutralizing antibody titers against SARS-CoV-2 on day 40

The geometric mean of the neutralizing titers among PPS cases on day 40 will be calculated. Additionally, the geometric mean ratio of the neutralizing titer on day 40-post immunization with the second dose of BNT162b2 mRNA to the geometric mean value and the 95% confidence interval calculated in the previous study (“*Study on the changes in antibody titers by vaccination with a novel coronavirus vaccine*”) will be used; the geometric mean of the neutralizing titer on day 40 and day 0 will be calculated for the test drug-inoculated cases among the PSS cases.

Additionally, the geometric mean of neutralizing antibody titers in non-vaccinated subjects on day 40 will be calculated to compare with that in KD-414 inoculated subjects.

(2) IgG-S on days 7 and 40 post-inoculation
(3) IgG-N on days 7 and 40 post-inoculation
(4) IgM-S on days 7 and 40 post-inoculation
(5) Th1 and Th2 responses against SARS-CoV-2 on day 7 post-inoculation
(6) QuantiFERON SARS-CoV-2 test on days 7 and 40 post-inoculation

Similar analyses as in primary endpoint analysis and analysis of the secondary variables (1) will be performed for the above six items. However, for the Th1 and Th2 endpoints, the analysis will be performed on data collected on day 7 post-inoculation.

(7) Among the PPS cases, the number and percentage of COVID-19 cases in the vaccinated and unvaccinated groups will be calculated.

**Analysis of safety endpoints**

The following safety endpoints will be summarized for the safety analysis set:

(1) Compilation of the incidence of all adverse events occurring between day 0 and day 40, deaths due to adverse events, serious adverse events other than death, significant adverse events, high-severity (Grade 3 or higher) adverse events, and causal relationship to study drug
(2) Physician-reported outcomes
  - Incidence, severity, days to onset, duration, incidence by dose, and causal relationship to study drugs of specified local adverse events
  - Incidence, severity, days to onset, duration, incidence rate by dose, and causal relationship to study drugs of specified systemic adverse events
  - Incidence, severity, days to onset, duration, incidence by dose, and causal relationship to study drugs of unspecified adverse events
(3) Calculation of summary statistics for maximum body temperature from day 0 to day 6 post-inoculation for each test drug
(4) Subject-reported outcomes

The following analyses will be conducted for the outcome measures in the health observation diary completed by the study subjects.

- The number and percentage of patients with symptoms of redness, swelling, induration, and pain during the 7-day period. A summary statistic is calculated for the maximum value in the case with the maximum diameter reported for redness and induration.
- In case of subjective symptoms and objective findings, the number and percentage of subjects with symptoms in the 7-day period will be calculated. In addition, a contingency table will be generated for the most common symptoms throughout the 7-day period.
- The number and percentage of subjects who used antipyretics will be calculated.

**Interim analysis**

No interim analysis will be performed in this study.

**Procedures for changing the original analysis plan**

If there are changes from the original statistical analysis plan, the study plan or statistical analysis plan should be revised and explained in the clinical study report.

**Final analysis**

Analysis will be performed after fixation of the case for which data are available. The statistical analysis manager will produce the analysis report and submit it to the principal investigator.

Once the data until day 40 are fixed, they will be analyzed. When a booster vaccination at public expense is implemented, data until day 40 will be collected again and analyzed after data fixation.

**Adverse events and diseases**
**Definition of adverse events**

An adverse event is an unfavorable symptom, sign, disease, or laboratory abnormality in the study subject, which may or may not be related to the test vaccine. Adverse events are defined as those occurring on or after day 0 (day of vaccine administration).

**Definition of disease**

The adverse events suspected to be attributable to the test vaccine or procedure are classified as “diseases.” The disease should be related to the drugs or procedure used in the study. The investigator or sub-investigator will determine the causal relationship based on the evaluation shown in “Causal relationship between KD-414 and adverse events”, below.

**Defining serious adverse events**

Serious adverse events are defined by the investigator or sub-investigator (hereafter referred to as the “investigator” or “sub-investigator”) based on the following criteria:

1. Leading to death
2. Requiring admission to a medical institution or prolongation of hospital stay for treatment
3. Leading to disability
4. Serious according to 1–3
5. Congenital disease or abnormality in later generations

Additionally, adverse events that are non-fatal, non-life-threatening, and do not require hospitalization may be considered “serious”. Serious adverse events are those that are judged to be important medical events that may put the subject at risk or require treatment or surgery to avoid the occurrence of the event. However, hospitalization for examination purposes and prolongation of hospitalization for examination purposes scheduled before participation in the study are not considered adverse events.

**Measures to be taken in case of adverse events**

(1) In case of an adverse event, the investigator will consider necessary medical treatment to ensure the safety of the subject
(2) The investigator should inform the subject when medical treatment is required.
(3) The investigator will confirm the resolution or stabilization of adverse events.

**Causal relationship between KD-414** **and adverse events**

The causal relationship between all adverse events and KD-414 will be determined by the physicians enrolled in the study. The judgment should be based not only on the temporal relationship with the inoculation of KD-414 but also on the underlying disease course, complications, concomitant medications, research procedures, accidents, and other external factors. Causality will be judged and recorded according to the following criteria:

**It is reasonable or possibly reasonable to attribute the adverse events to the research procedures or KD-414 inoculation.**

Undeniable causal relationship: the decision should be made according to the following criteria irrespective of whether adverse events are known to occur in the clinical study or during vaccination:

➢ There is a temporal relationship between studies.
➢ No other cause is shown, and a causal relationship to KD-414 cannot be ruled out.

- Not related; judged according to the following criteria:

➢ Not reasonable to attribute the adverse events to research procedures or KD-414 inoculation
➢ Show no temporal relationship
➢ Can indicate other causes

**Assessment of adverse events**
**Evaluation of adverse events**

The presence or absence of abnormal findings will be determined at each examination during the period from post-vaccination to Visit 2 (post-hoc observations). The details of the event judged as “abnormal” will be recorded in the Case Report Form as an adverse event.

**To determine adverse events at the inoculation site**

The details of the local reaction observed during the period from post-vaccination to Visit 3 (post-hoc observations) will be recorded in the Case Report Form as an adverse event.

**Evaluation of adverse events listed in the health diary**

The contents of the health diary during the period from post-vaccination to Visit 3 (post-hoc observation) will be reviewed and the adverse events will be assessed based on medical judgment.

**Severity classification of adverse events**

The investigator or sub-investigator will determine and record the severity (severity) of each adverse event. Specific local adverse events and specified systemic adverse events will be judged using the Guidance for Industry Toxicity grading scale for healthy adult and adolescent volunteers enrolled in preventive vaccine clinical trials [11] and recorded in grades 0–4. Unspecified adverse events are categorized according to their impact on daily activities as Grade 1 (those that do not interfere with daily activities), Grade 2 (those that interfere with daily activities), Grade 3 (those that prevent performance of daily activities), and Grade 4 (potentially life-threatening).

**Severity classification of specified local adverse events (inoculation site)**

The investigator or sub-investigator will determine the severity of local adverse events based on the “Classification of severity of local reactions (inoculation site)” definition in Table 6.

**Severity classification of specific systemic adverse events**

The investigator will determine the severity of specific systemic adverse events for the subject based on Table 7 and Table 8.

**Predictive judgment**

The predictability of adverse events will be determined based on the description in the Investigator’s Brochure. Adverse events not listed in the Investigator’s Brochure and those listed but with inconsistent severity or frequency are considered unknown adverse events.

**Procedures for collecting, recording, and reporting information on diseases**

The investigator or sub-investigator will record all adverse events in study subjects during the study period to determine a causal relationship.

In the event of an illness (a serious adverse event) in the study subject, the NCGM will promptly report the event to the supervisor of the participating medical organization and NCGM Clinical Research Safety Management Office irrespective of its relationship with the administered vaccine. NCGM reports the event (a disease) to the NCGM Clinical Research Review Board. Adverse events suspected to be attributable to the conduct of clinical research are classified as “diseases.” Events attributable to the study should be related to the drug used in the research or the research procedure. Detailed reporting procedures shall be according to the “Procedures for response to adverse events/diseases.”

**Duration of adverse event information collection: 40 Days**
**Reporting of diseases to the certified Clinical Research Review Board (research using unapproved or off-label drugs, etc.; Table 9)**

**Table 9:** Reporting of diseases to the certified Clinical Research Review Board.

Studies Classification	Predictability	Severity of Illness	Reporting Deadline
Clinical research using unapproved or off-label drugs, etc.	Not possible	Death	7 days
		Diseases that may lead to death	
	Possible	Death	15 days
		Diseases that may lead to death	
	Not possible	Diseases requiring admission to a medical institution or prolonged hospital stay for treatment	15 days
		Disorder	
		Diseases that may lead to disability	
		Serious illnesses accordance to the above and illnesses that may lead to death	
		Any congenital disease or anomaly in the offspring of a treated patient.	
Occurrence of diseases suspected to be attributable to the conduct of clinical research (other than those reported above)			Periodic Report

① The investigator should report a disease that may lead to death to the NCGM Institutional Review Board, the MHLW, and the Pharmaceuticals and Medical Devices Agency (PMDA) after reporting it to the supervisor of the participating medical institution and NCGM Clinical Research Safety Management Office within 7 days. Forecasts should be reported to the NCGM Clinical Research Review Board after reporting them to the administrators and NCGM Clinical Research Safety Management Office of the participating institution within 15 days.

Additionally, the following diseases that cannot be predicted are reported to the supervisor of the participating medical organization and NCGM Clinical Research Safety Management Office within 15 days before reporting them to the NCGM Clinical Research Review Board, the MHLW, and PMDA:

② Diseases requiring admission to a medical institution or prolonged hospital stay for treatment
③ Diseases that may lead to disability
④ Serious diseases according to ①–③.
⑤ Any congenital disease or anomaly in the offspring of a treated patient.

Furthermore, events that are non-fatal, non-life-threatening, and do not require hospitalization may be considered “serious” if they are judged to be an important medical event that may place the subject at risk, or if treatment or surgery is required to avoid the occurrence of the event. However, hospitalization for examination purposes, and prolongation of hospitalization for examination purposes scheduled before participation in the study, are not considered serious adverse events.

Disease reports to the MHLW will be prepared from disease reports of jRCT (Clinical Research Protocol and Research Outline Disclosure System).

The occurrence of diseases suspected to be attributable to the study procedures (excluding diseases subject to the above reports) will be reported at the time of periodic reporting by the NCGM Review Board.

**Observation period of study subjects after the outbreak of disease**

Observations of the study subjects after the occurrence of adverse events will be followed up until the alleviation of the adverse event or the investigator (or sub-investigator) judges that no follow-up is required. Detailed follow-up procedures shall be performed according to the “Procedures for response to adverse events/diseases.”

**Viewing source documents**

The investigator and the institution shall ensure direct access to all clinical research-related records, such as source documents during clinical research-related monitoring, auditing, and NCGM Clinical Research Review Board and regulatory review.

**Quality control and assurance**
**Method of monitoring**

The investigator will prepare a written procedure for monitoring each study protocol and conduct monitoring as specified in the relevant procedure and study protocol.

Personnel involved in clinical research should not be monitored for tasks directly responsible for such individuals. Personnel involved in monitoring will report the results of such monitoring to the investigator.

**Auditing method**

The investigator shall prepare a written audit procedure for each study plan and conduct an audit as specified in the relevant procedure and study protocol. Individuals involved in clinical studies must be subjected to audits, whereas those involved in their monitoring should not be audited. Personnel involved in the audit will report the results of the audit to the investigator.

**Method of data management**

Data management will be performed by the personnel of the JCRAC data center.

Data collection will be performed using the EDC system as an electronic case report form (eCRF) and management tool.

Fixed data will be provided to the analysis manager after data fixation at the data center. Details are specified in the data management plan.

**Ethical considerations**
**Compliance with laws and regulations**

This study will be conducted according to the ethical principles, Clinical Trials Act (Act No. 16 of 14 April 2017), related notifications, and the study protocols stipulated in the Declaration of Helsinki. Materials stipulated in the law, including research protocols and protocols, will be discussed and approved by the Certified Clinical Research Review Board. Subsequently, the investigator will complete enrollment in the jRCT with permission from the administrator of the participating institution before initiating the study. The same procedures will be followed if there are any changes in these materials before implementing the amendments. The investigator will ensure that all researchers complete educational training on research ethics and other necessary skills at their respective participating medical institutions and will continue to receive educational training during the study.

**Benefits and burdens incurred by clinical research subjects, anticipated disadvantages, as well as measures to minimize them**

Participation in this study will determine antibody titers before and after immunization with KD-414. Immunization with KD-414 is expected to elicit antibody responses against SARS-CoV-2 and may increase the likelihood of preventing infection. However, it is unclear whether the antibodies can completely prevent COVID-19.

As KD-414 is not approved and there is no adequate vaccination experience, unknown adverse events may occur. Nonclinical studies and Phase I/II studies conducted with KD-414 have not revealed any safety concerns.

No adverse reactions to KD-414 have been reported. Pfizer comparatively analyzed the frequencies of various adverse events between individuals who received the third booster dose (*n* = 289) and those who received two doses (*n* = 2682) (fever, 8.7% vs. 16.4%; malaise, 63.7% vs. 61.5%; headache, 48.4% vs. 54.0%; and myalgia, 39.1% vs. 39.3%). Studies have also reported similar or improved tolerability of vaccinations with different novel coronavirus vaccines.

Based on the results of this study and the prevalence of COVID-19, further booster doses will be considered.

**When there is a possibility that research may provide important information on the health of subjects or genetic characteristics that can be inherited by their offspring, as a result of the conduct of the research, the handling of the results of research on study subjects including incidental findings which are findings outside the main objectives of the research).**

Not applicable

**Handling personal information**

Personnel involved in this study (including external parties) should comply with the Act on the Protection of Personal Information (promulgated on 30 May 2003, Law No. 57) and related notices applicable to the protection of personal information of study subjects. Personnel involved in this study should not acquire personal information by false or fraudulent means or reveal the personal information without justifying reasons even after the personnel leave their jobs and strive to protect the personal information and privacy of the study subjects.

Personnel involved in this study should not handle personal information acquired in this study beyond the scope of the informed consent provided by the subjects.

The investigator must identify the purpose of using personal information and maintain accurate and up-to-date personal information within the range necessary to achieve the objectives. Additionally, necessary measures should be taken to prevent the leakage, loss, or damage of personal information and appropriately manage personal information. The methods for these measures shall be specifically stipulated by the Implementation Regulations.

**Anonymization methods and management**

The information of study subjects should be identified using a novel unique number granted by the personnel involved in the study at the time of enrollment (Clinical Research Subject Identification Code) without the information that will lead to the identification of a particular individual.

The investigator shall prepare a correspondence table for the name and identification code of the study subject and store it appropriately in a lockable location within the participating medical organization.

**Handling and storage of records (including data)**
**If the purpose of use includes the provision of samples or information to another institution.**

Research institute: KM Biologics Co., Ltd.

Sample/information: The sera from day 29 (day 7 post-second dose) stored in previous studies and those from day 0, 7, and 40 post-inoculation in this study will be provided to investigate the neutralizing antibodies.

**Storage and disposal of samples and information**
**Retention of records**

The investigator will retain the records of this study along with the following documents:

(1) Identification code list for subjects of clinical research, research protocol, protocol, explanation to subjects of clinical research and documents related to their consent, clinical study report, other clinical research law, documents prepared by the investigator according to the regulations for enforcement, and their copies.
(2) Documents related to review opinions received from NCGM Clinical Research Review Board
(3) Monitoring and audit documentation
(4) Source documents
(5) Contract for conducting this study
(6) Prepared documents and records that describe the outline of the drugs used in this study
(7) Other documents required to conduct this study

**Retention period and location of records**

The investigator will retain the records appropriately in a key archive within the institution for five years from the date of completion of the study (the day the summary of the clinical study report was published to jRCT).

In case these materials are entrusted to an outside contractor and stored, arrangements shall be made in the contract so that they can be periodically monitored and controlled under appropriate conditions during the specified storage period.

**Method for sample and information** **disposal**

The personal information must be carefully handled when discarding specimens and information related to this study. The samples used in this study should be discarded appropriately according to the in-house procedures. The information must be discarded by placing the paper medium on a shredder. The electronic recording medium must be discarded in a non-readable state. The file in the personal computer should be completely deleted such that it cannot be reproduced.

**Payments and compensation related to the conduct of clinical research**
**Presence and content of cost and burden reduction expenses for study subjects**

KD-414 to be administered in this study will be provided free of charge by companies. The cost of testing will also be covered by the research costs to avoid a cost burden to the subjects. For the research subjects, up to JPY 60,000 will be paid to mitigate the cost burden (JPY 20,000 per blood sampling for KD-414 vaccines and JPY 2000 per blood sampling for non-vaccines). For post-booster vaccination at public expense, up to JPY 6000 will be paid as a burden reduction cost (JPY 2000 per blood sampling).

**Presence or absence and content of insurance coverage**

To compensate for the loss incurred by the research subject due to health hazards, the research representative physician will have clinical research insurance with the following compensation content. The study subject will be compensated according to the clinical research insurance payment conditions.

▪ Compensation for death or sequelae disability of study subjects
▪ Medical expenditures and medical benefits required to treat the health hazards of research subjects

Additionally, the principal investigator and the sub-investigator must also have physician’s liability insurance in preparation for health damage to the study subjects resulting from the usual range of medical practices in this study.

**Existence and content of compensation other than insurance**

In the event of any health hazard to a study subject due to the implementation of this study, the investigator, sub-investigator, and participating medical institution shall take the necessary measures, such as the provision of medical treatment so that the study subject can receive the appropriate diagnosis, treatment, and necessary measures immediately.

**Publication of information on clinical research**
**Method of publication**

During this study, jRCT should document the items required by the World Health Organization for publication and other items that contribute to ensuring the transparency of the clinical research process and the selection of the volunteers to participate in clinical research and publish these items. The study will be initiated after jRCT publication. However, changes will be made to the protocol and the information will be updated according to the progress of the study. If a primary endpoint report or clinical study report is prepared, a summary of the primary endpoint report or clinical study report will also be published. The articles will also be presented.

**Content of publications and agreements regarding the timing and results of clinical research with the marketing authorization or marketing authorization of pharmaceuticals that have received grants, if any.**

The investigator, statistical analysis manager, and GLIDE office will select the authors after consultation and report them to academic societies and/or journals. Based on the contract with the company, prior notice shall be given to KM Biologics, Ltd. before the announcement of the results. The results shall be publicly announced after due consultation where necessary. However, the company is not involved in the interpretation of the results of this study and cannot refuse to provide public consent without justification. The results will be reported when KM Biologics Co., Ltd. wishes to disclose them.

**Duration of clinical research**
**Planned study period:** jRCT publication date to 31 March 2023 (study completion date: date of publication of the summary of the clinical study report in jRCT)
**Explanation and obtaining informed consent for subjects in clinical research**

The investigator (or sub-investigator) will provide full explanations using documentary information before participation in the study and obtain voluntary consent for participation. The purpose and significance of the clinical research and the methods and duration of the clinical research will be explained.

**Preparation of explanatory documents, written consent forms, and consent withdrawal forms**

The investigator will prepare a single form of written information, informed consent form, and consent withdrawal form for each study protocol and obtain approval from the NCGM Clinical Research Review Board.

Plain language will be used so that the study subjects can easily understand the contents.

**Explanation items**

Explanatory matters include the matters provided for in Article 46 of the following regulations.

(1) The name of the specified clinical research to be conducted, the approval received by the manager of the participating medical organization for conducting the specified clinical research, and the implementation plan submitted to the Ministry of Health, Labor and Welfare.
(2) The names and titles of the participating medical organizations and the investigators.
(3) Reasons for selecting subjects for specific clinical studies
(4) Anticipated benefits and disadvantages of conducting specific clinical studies
(5) The option of voluntarily refusing participation in specific clinical research.
(6) Matters concerning withdrawal of consent
(7) Denial of participation in specified clinical research or withdrawal of consent does not result in unfavorable handling.
(8) Method of disclosing information on specific clinical research
(9) The availability or browsing of research protocols and other data related to the conduct of specific clinical research and the methods of obtaining or browsing them in response to the request of the test subjects or their representative (hereafter referred to as the target person of specified clinical research, etc.).
(10) Items related to the protection of personal information of study subjects
(11) Method of storage and disposal of samples.
(12) Status of involvement in specified clinical research as prescribed in the items of Article 21, paragraph (1) of the Ordinance on Specified Clinical Research: Matters concerning a conflict of interest
(13) System for responding to complaints and inquiries
(14) Items related to costs related to the conduct of specified clinical research
(15) Presence and content of other treatment and comparison with expected benefits and adverse benefits from other treatment
(16) Matters related to compensation and provision of medical care for health hazards from conducting specific clinical research
(17) Items to be reviewed by the NCGM Clinical Research Review Board concerning opinions for specified clinical research and other items related to the Accredited Clinical Research Review Board related to such specified clinical research.
(18) Other necessary items related to the conduct of specific clinical research

**Method of obtaining consent for clinical research subjects**

The investigator or sub-investigator will provide an easy-to-understand explanation on clinical research using an explanatory document with permission from the certified clinical research review board before obtaining written informed consent to participate in the study. The study subjects are allowed to ask questions and provided with time to decide on participation in the study. The investigator or sub-investigator will satisfactorily answer the questions. Additionally, the investigator or sub-investigator will explain that the consent for study participation also includes the consent for direct access to the medical records of the study subject during monitoring, audits, certified clinical research review board meetings, and regulatory surveys. The investigator or sub-investigator and consenting clinical research subjects will sign the informed consent form along with the date. After obtaining the informed consent, the investigator or sub-investigator will retain the original copy of the informed consent form and provide a copy of the informed consent form to the study subjects. The investigator or sub-investigator will document that a copy of the information and informed consent form has been provided to the study subject (original of the consent form, medical record, etc.).

**Appointment of a proxy consenter and obtaining consent from the proxy consenter**

Not applicable

**When minors are included in clinical research**

Not applicable

**Secondary use of samples and information**
**The possibility of secondary use of specimens and information or providing them to other research institutions**

As the specimens and information of the study participants are invaluable, they may be used for future studies (including human genome/gene analysis studies) that are not identified at the time of receiving consent from the study subjects and may be provided to other research institutions or companies around the country.

When existing data are stored and archived samples are used for new research, a new study protocol, will be provided to the Ethics Review Board and used after approval. Additionally, a public information document is prepared by the opt-out procedure to ensure the opportunity for subjects to refuse to participate in the research. Existing data and stored samples can be provided to researchers or companies in other facilities for use within the scope of informed consent. Measures must be undertaken to prevent the identification of individuals without providing anonymization response tables.

In addition, data and stored samples will be transferred to REBIND at any time to collect information, excluding the remaining sample and personal information after the completion of this study. Specimens and data transferred to REBIND may be transferred to other agencies (including companies).

※REBIND

Repository of Data and Biospecimen of INfectious Disease (REBIND)

A public biobank project was conducted by the Ministry of Health, Labor, and Welfare. See the website for more information on REBIND. <https://rebind.ncgm.go.jp/> (accessed on 18 June 2022).

**Procedures for secondary use of samples and information**

When archived specimens and existing data are used for new research, a new study protocol will be provided to the Ethics Review Board and used after approval. Additionally, the new protocol should be used only for research contributing to infectious diseases. In case existing data and samples are provided to researchers at other institutions, they shall be used only for research contributing to infectious diseases and provided within the scope of informed consent. Anonymized response tables should not be provided. Measures should be taken to prevent the identification of individuals.

**Response to consent** **withdrawal**

In case the study subjects withdraw their consent for participation in the study, the investigator or sub-investigator will consult with them to confirm that they are not reluctant to withdraw consent, confirm the reason for the withdrawal of consent, and explain the examinations and tests specified after consent withdrawal. The subjects will be requested to fill in the consent withdrawal form or the consent withdrawal will be recorded in documents, such as the medical record. In the event the study subjects who have withdrawn consent do not provide permission to use their samples and information, they will be excluded from the analysis.

When the results of the clinical research are already published or when medical devices are implanted in the body and cannot be easily removed, it is not possible to respond to some or all the consent withdrawals from the study subjects. In such cases, the investigator or sub-investigator will explain the reason to study subjects and arrive at an understanding.

Requesting the reasons for withdrawal of consent may lead to the retraction of the offer. Therefore, this should be addressed regardless of whether or not the reason for the offer was provided, except in cases where it interferes with ensuring the safety of the study subjects.

**Handling of new information that influences the consent provided by the study subjects**

The investigator or sub-investigator should promptly inform the study subjects about new important information and obtain their consent to continue their participation in the research. Additionally, the new information and informed consent form will be revised to obtain approval from the NCGM Clinical Research Review Board.

**Revision of documents and informed consent forms (entry of the revised version number and date of preparation)**

In case the study-related information and informed consent form are revised, the investigator or sub-investigator will promptly explain the objectives of clinical research to the study subjects using the revised information and informed consent form and obtain written consent from the study subjects for continued participation in the study.

**Items required for conducting clinical research**
**Matters related to conflicts of interest**
**Names of organizations providing research funding (public research expenditures/companies)**

KM Biologics Co., Ltd. (project for emergency improvement of vaccine production systems) (contracted expense).

The test drugs were provided free of charge by KM Biologics Co., Ltd. Additionally, the research fund was used to sign a contract with CTD Co., Ltd. (Tokyo, Japan) for assisting with the administration of this study, and Accerise, Inc. (Tokyo, Japan). for monitoring, auditing, and performing statistical analysis. KM Biologics is not involved in the analysis of the results and will not influence the results or the publication of the findings.

**Conflicts of interest management**

Before the implementation of this study, the investigator or sub-investigator will prepare and review the conflicts of interest according to the provisions of the NCGM Conflict of Interest Management Committee (including the preparation of the Conflict of Interest Control Standards (Form A), Reports of Affiliated Industries (Form B (if occurring)), Reports of Investigator Conflict of Interest Self-Reports (Form C), and Conflict of Interest Control Plans (Form E)).

The principal investigator will submit the NCGM Conflict of Interest Management Committee review report to the NCGM Clinical Research Review Board for review and approval. Additionally, the principal investigator will continuously monitor, manage, and publish the conflicts of interest throughout the study period.

**Studies (such as life-saving clinical research in an emergency setting) for which obtaining consent** **is difficult**

Not applicable.

## 3. Discussion

The COVID-19 pandemic is currently ongoing, and there have been significant efforts in the development of COVID-19 vaccines. However, the neutralizing antibody titers in vaccinated individuals are reported to progressively decrease over time. Japanese pharmaceutical companies have published the results of Phase I and II studies on the safety and efficacy of different vaccines. Final clinical trials will be conducted with the aim of practical application by March 2023. Since mRNA vaccination is progressing around the world, it is necessary to evaluate the efficacy and safety of booster doses in individuals who have received two doses of mRNA vaccine in order to make effective use of inactivated vaccines in the future. There have been studies evaluating the progression of antibody titers with boosters with inactivated vaccines after initial immunization with inactivated vaccines and with boosters with mRNA vaccines, but few studies have evaluated boosters with inactivated vaccines after initial immunization with mRNA vaccines [12,13]. This is the first study of booster immunization with KD-414 developed in Japan.

This manuscript describes the protocol for a study that aims to examine the effect of a booster dose with KD-144, a novel inactivated Japanese vaccine, on antibody titers. A previous study comparing the efficacy, side effects, and seroconversion of various vaccine formats against SARS-CoV-2, including inactivated, recombinant, mRNA, and nano-particle-based vaccines suggested that inactivated vaccines have fewer side-effects and similar seroconversion compared to other types of vaccines [14]. Furthermore, heterologous prime-boost vaccine strategies have been shown to result in improved immunogenicity, reactogenicity, safety, effectiveness and flexibility, as well as to mitigate against intermittent supply shortages [15,16].

There are some limitations to this protocol. First, it is designed as a single-arm study with no randomization. Second, the follow-up period will be relatively short (40 days) because the COVID-19 vaccination at public expense commences 42 days after KD-414 vaccination. Third, the definition of an ‘appropriate’ amount of KD-414 vaccination is unclear; the dose used in this study was determined based on the dose of the first and second shots of KD-414 in Phase I and II clinical trials.

## Figures and Tables

**Table 1:** Study calendar (KD-414 group).

Visit			Visit 1			Observation period	Visit 2	Visit 3
			KD-414 inoculation				Post hoc observation	Post hoc observation
Expiration day (Day) * 1		Obtaining written consent	Day 0			Days 1–7	Day 7	Day 40
[Acceptable range]			Pre	Inoculation	After		[±1 day]	[±1 day]
Medical institutions	KD-414 inoculation			◯				
	Examination		◯		△ * 2		◯	◯
	Body temperature measurement		◯					
	Volume of blood sampling		17 mL				22 mL	17 mL
	Immunogenicity		◯				◯	◯
Home	Observation of adverse events					◯	◯	◯
	(Health diary entries)							

◯: Essential, △: Occurrence of an adverse event. * 1: The day of immunization with KD-414 will be considered day 0. * 2: Performed at 15–30 min post-vaccine inoculation.

**Table 2:** Study calendar (KD-414 non-vaccinated).

Visit			Visit 1	Visit 2	Visit 3
Expiration Day (Day) * 1		Obtaining written consent	Day 0	Day 7	Day 40
[Acceptable range]				[±1 day]	[±1 day]
Medical institutions	Volume of blood sampling			19 mL	14 mL
	Immunogenicity			◯	◯

◯: Essential. * 1: The day of KD-414 inoculation is considered day 0.

**Table 3:** Study calendar (public cost booster).

Visit		Visit 1	Observation period	Visit 2	Visit 3
Expiration Day (Day) * 1		Day 0	Day 1–7	Day 7	Day 40
[Acceptable range]				[±1 day]	[±1 day]
Medical institutions	Public cost vaccination	◯			
	Examination	◯		◯	◯
	Body temperature measurement	◯			
	Volume of blood sampling	17 mL		22 mL	17 mL
	Immunogenicity	◯		◯	◯
Home	Observation of adverse events	◯	◯	◯	◯
	(Health diary entries)				

◯: Essential. * 1: The day of public cost vaccination date was considered day 0.

**Table 4:** Evaluation criteria for determination by reagents.

Outcome		Determination	
		Positive	Negative
①	Determination of neutralizing antibodies against live SARS-CoV-2 virus (fold)	≥40	<40
②	Antibody (IgG) titers against the viral nucleocapsid protein (Index or S/C)	≥1.40	<1.40
③	Antibody (IgM) titers against viral spike proteins (Index or S/C)	≥1.00	<1.00
④	Antibody (IgG) titers against viral spike proteins (AU/mL)	≥50.0	≥50.0

**Table 5:** Power calculations.

	Detection Power 80%	Detection Power 85%	Detection Power 90%
SD 0.35	34	39	45
SD 0.40	44	50	58

**Table 6:** Classification of severity of local reactions (inoculation site).

**At the Injection Site; Local Reaction**	**Mild** **(Grade 0)**	**Mild** **(Grade 1)**	**Moderate (Grade 2)**	**Severe** **(Grade 3)**	**Life Threatening; to Be Feared** **(Grade 4)**
Pain	-	Prevent activity	Repeated use of non-narcotic analgesics; >24 h or interfere with daily activity	Use of narcotic analgesics; interferes with daily activities	Emergency room visits or hospitalization
Erythema/redness *	<2.5 cm	2.5–5 cm	5.1–10 cm	>10 cm	Necrotizing or exfoliative dermatitis
Indurated **	<2.5 cm	2.5–5 cm; does not interfere with activity	≥5.1–10 cm or interferes with activity	>10 cm daily; interferes with daily activities	Necrosis
Swelling **	<2.5 cm	2.5–5 cm; does not interfere with daily activity	≥5.1–10 cm or more or interferes with daily activity	>10 cm daily; interferes with daily activities	Necrosis

* The measured local response is graded by its maximum diameter, and measurements are recorded. ** They are evaluated and graded using the Functional Scale, as well as measured values.

**Table 7:** Severity classification of systemic reactions (1).

Vital Sign *	Mild (Grade 0)	Mild (Grade 1)	Moderate (Grade 2)	Severe (Grade 3)	Life-Threatening; to Be Feared (Grade 4)
Fever (°C) **	37.5–37.9	38.0–38.4	38.5–38.9	39.0–40	>40

* All vital signs should be measured at rest. ** Oral temperature; no recent hot or cold drinks or smoking.

**Table 8:** Severity classification of systemic reactions (2).

Whole-Body	Mild (Grade 1)	Moderate (Grade 2)	Severe (Grade 3)	Life-Threatening (Grade 4)
Nausea and vomiting	No interference with activity or vomiting 1–2 times within 24 h	Any interference with activity or vomiting ≥2 times within 24 h	Interferes with daily activities; requires intravenous fluids in the outpatient setting	Emergency room (ER) visits or hospitalization for hypotensive shock
Headache	No interference with activity	Use of non-narcotic analgesics for more than 24 h, causing some interference with activity	IMPORTANCE: Use of narcotic analgesics Interferes with daily activities	ER visits or hospitalization
Fatigue (Malaise)	No interference with activity	Cause some interference with activity	IMPORTANCE: Impairing daily activities	ER visits or hospitalization
Myalgia	No interference with activity	Cause some interference with activity	IMPORTANCE: Impairing daily activities	ER visits or hospitalization

## Data Availability

Data sharing is not applicable to this article as no datasets were generated or analyzed during the current study.

## Abbreviations

CDC	Centers for Disease Control and Prevention
COVID-19	coronavirus disease 2019
NCGM	National Center for Global Health and Medicine
REBIND	Repository of Data and Biospecimens of INfectious Disease
